# Invasion of dwarf bamboo into alpine snow-meadows in northern Japan: pattern of expansion and impact on species diversity

**DOI:** 10.1002/ece3.9

**Published:** 2011-09

**Authors:** Gaku Kudo, Yukihiro Amagai, Buho Hoshino, Masami Kaneko

**Affiliations:** 1 Faculty of Environmental Earth Science, Hokkaido UniversitySapporo, Hokkaido 060-0810, Japan; 2 Department of Biosphere and Environmental Sciences, Rakuno Gakuen UniversityEbetsu 069-8501, Japan

**Keywords:** Alpine vegetation, global warming, remote sensing, *Sasa kurilensis*, snowmelt, soil moisture

## Abstract

Recently, a dwarf bamboo species,*Sasa kurilensis*; Poaceae, has invaded into alpine snow-meadows in the wilderness area of the Taisetsu Mountains, northern Japan. This dwarf bamboo species has a wide distribution range from lowland to alpine sites of snowy regions. Because of the formation of dense evergreen culms and an extensive rhizome system, other plants are excluded following invasion by this dwarf bamboo, resulting in low species diversity. Dwarf bamboo originally inhabited the leeward slopes of alpine dwarf pine (*Pinus pumila*) clumps in alpine regions. During the last 32 years, however, dwarf bamboo has expanded its distribution area by up to 47% toward snow-meadows, especially on southeastern facing slopes. This rapid change may be related to the decrease in soil moisture and expansion of the annual growing period caused by the recent acceleration of snowmelt time. A multiyear census revealed that the density of bamboo culms increased 30–150% during 2 years, and the annual expansion of bamboo rhizomes was 39 cm on average. In addition to the expansion of bamboo clumps by vegetative growth, the possibility of migration by seed dispersal was also suggested by a genet analysis. With the increase in culm density, the species richness of snow-meadow vegetation decreased to less than one-quarter of the original level due to intense shading by dwarf bamboo. The rapid vegetation change in these almost pristine alpine environments isolated from the human activity implies that global climate change already influences the alpine ecosystem.

## Introduction

Alpine and high-latitude ecosystems are most susceptible to climate change, and rapid vegetation changes have been reported in several tundra and alpine regions (Hughes 2000; IPCC 2001). In Alaskan tundra, shrubs and graminoids increased in biomass, resulting in decreased species diversity in wet tundra during the last 20 years (Chapin et al. 1995). Expansion of shrubs has been reported in several tundra and alpine regions (Sturm et al. 2001, 2005; Dullinger et al. 2003; Tape et al. 2006; Knapp et al. 2008) because of warming in winter and spring, early snowmelt, or soil desiccation. In alpine regions, the timberline has moved upward in many regions, although this is dependent on the regional vegetation types (Körner and Paulsen 2004; Harsch et al. 2009). The upper distribution limit of alpine plants has shifted upward in the European Alps (Grabherr et al. 1994; Pauli et al. 2007) but the responses of species are individualistic. These studies indicated that the impact of global warming on tundra and alpine vegetation is highly specific to regions and species. This has been supported by warming experiment in tundra and alpine regions conducted over a wide range of geographic regions (Arft et al. 1999; Walker et al. 2006).

Effects of vegetation change on ecosystem function and species diversity are highly dependent on the dynamics of a few key species (Chapin and Shaver 1985; Klanderud 2008). For instance, the expansion of dwarf birch (*Betula nana*) has caused a decline of species diversity in experimental plots in Alaska (Chapin et al. 1995), reportedly mainly due to the high developmental plasticity of*B. nana*and thus a flexible growth strategy (Bret–Harte et al. 2001). Climatic amelioration may change the relationship among existing species within communities from facilitative to competitive (Callaway et al. 2002), by which changes in species composition and richness are accelerated by competitive exclusion. Invasion of shrubs and trees especially causes a serious impact on alpine and tundra vegetation because tall aboveground structure may depress the growth of other plants with shorter structures because of shading. In addition, an increase in biomass may change edaphic environments, such as soil water conditions and nutrient cycling. Therefore, the dynamics of key dominant species in each ecosystem should be clarified to predict the ecological impact of climate change on regional ecosystems.

Dwarf bamboo shrubs (*Sasa*spp., Poaceae) are common and predominant plants in northeastern Asia (Numata 1970) ranging from costal to alpine regions. Because of dense culm production and an extensive rhizome network, dwarf bamboo often predominates over large area. In Hokkaido, northern Japan, dwarf bamboo was estimated to occupy 89% of forested areas and accounts for 28% of the woody biomass (Toyooka et al. 1983).*Sasa kurilensis*Makino et Shibata is the most common dwarf bamboo in snowy regions. Its plant height sometimes attains over 2 m, and its aboveground biomass reaches 3 kg/m^2^ (Yajima et al. 1997). This species is evergreen, and individual culms live for several years. Because of the strong shading effects by dense culms, dwarf bamboo often prevents the regeneration of cool-temperate forests in northern Japan (Nakashizuka 1988; Hiura et al. 1995; Takahashi 1997). Above timberline,*S. kurilensis*is commonly distributed around clumps of alpine dwarf pine (*Pinus pumila*). Shelter effects by*P. pumila*cause snow accumulation during the winter that protects dwarf bamboo from low temperatures. On the other hand, snowmelt at bamboo habitats should not be later as between early to mid-June. Thus, the distribution of dwarf bamboo in alpine regions is highly influenced by snow conditions.

**Figure 1:**
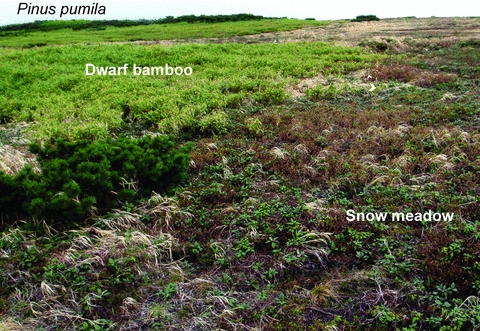
Advancing front of dwarf bamboo (*Sasa kurilensis*) in alpine snow-meadows in the Taisetsu Mountains. Although dwarf bamboo basically inhabited the vicinity of*Pinus pumila*clumps in the alpine area, it is expanding toward moist snow-meadow sites. Photograph by G.K. in late September 2009.

Recently, snowmelt time in alpine snow-meadows has become earlier in the Taisetsu Mountains, Hokkaido (Kudo and Hirao 2006). Along with the changes in snowmelt patterns, the distribution of dwarf bamboo has expanded from the vicinity of*P. pumila*clumps toward later snowmelt sites in the Taisetsu Mountains (G. Kudo, personal observation: Fig. 1). Alpine plants have adapted to high irradiation and are sensitive to low-light conditions (Billings 1974; Körner 2003). Expansion of dwarf bamboo, therefore, could cause a serious impact on the distribution of alpine plants and the species diversity of alpine vegetation. To predict the ecological impact of dwarf bamboo expansion on alpine ecosystem, understanding of the regeneration system of dwarf bamboo is necessary. Dwarf bamboo species are long-lived, monocarpic plants, and regeneration by sexual reproduction had been thought to be effective after mass flowering events (Numata 1970; Makita et al. 1988, 2004). However, recent studies on clonal identification of dwarf bamboo species revealed that not only vegetative expansion of clonal clumps but also sporadic sexual reproduction occurs in forest zones (Yamazaki and Nakagoshi 2005; Kitamura and Kawahara 2009). However, evidence of sexual reproduction in alpine zones has not been forthcoming.

In this study, (1) we surveyed the increasing area of dwarf bamboo and the invasion pattern into alpine snow-meadows during the last 32 years based on an analysis of aerial photographs, (2) quantified the biomass and year-to-year growth pattern using field censuses, (3) evaluated the impact of dwarf bamboo invasion on species diversity, and (4) predicted the reproduction mode based on genotype identification using a molecular technique.

## Materials and Methods

### Study site

**Figure 2:**
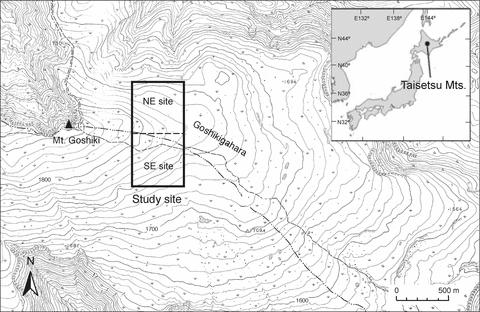
Location of the study site in the Taisetsu Mountains. A 500 × 1000 m^2^ plot was set on an east-facing gentle slope that was divided into NE site and SE site.

This study was conducted at an alpine snow-meadow named Goshikigahara located in the central part of the Taisetsu Mountains (43°33–34′N, 142°53–55′E) in Hokkaido, northern Japan (Fig. 2). Large parts of this mountain region are designated as a special protection area, where the effects of human activity and grazing by livestock on the ecosystem have been minimal. The summits of the Taisetsu Mountains are at about 1900- to 2100-m elevation, and the timberline is located at around 1500- to 1600-m elevation. Goshikigahara is situated on a gentle, east-facing slope at 1700- to 1850-m elevation (Fig. 2). Because of strong northwest winds during the winter season, large snowfields are formed on southeast-facing slopes in this region. Snowmelt usually occurs in early to mid-May on the ridges and upper slopes where alpine dwarf pine (*P. pumila*) dominates, while snowmelt occurs in mid- to late July at the bottom of the snowbed. Dwarf bamboo (*S. kurilensis*) is distributed between the*P. pumila*clumps and snow-meadow communities (Fig. 1). Annual mean air temperature at 1700-m elevation at a site 3 km from the research site was –2.0°C; temperature during the summer season was 2.9°C in May, 8.5°C in June, 11.6°C in July, 12.5°C in August, and 7.8°C in September (mean values of 2002–2010). Precipitation during the summer season was 127 mm in June, 225 mm in July, 331 mm in August, and 208 mm in September (mean values of 2002–2007).

Because of the abundant lingering snow and wet summer weather, humid soil conditions are retained during the summer, and alpine snow-meadows composed of herbaceous species, such as*Anemone narcissiflora*,*Trollius riederianus*,*Saussurea riederi*, and*Ranunculus acris*, are common on southeast facing slopes.

### Remote sensing analysis

The distribution of dwarf bamboo clumps was observed within a 500 × 1000 m^2^ area in the central part of Goshikigahara (Fig. 2). This area was divided into a northeast-facing slope (hereafter NE site) and a southeast-facing slope (hereafter SE site). The dynamics of dwarf bamboo were compared between 1977 and 2009 using aerial photographs provided by the Geographical Survey Institute, the Government of Japan (for the 1977 photograph) and Photec Co. Ltd. (Sapporo, Japan: for the 2009 photograph). Clear color photographs taken in September enabled us to discriminate readily clumps of dwarf bamboo (in bright green) and*P. pumila*(dark green) from other vegetation. Geographic information of these photographs was digitalized after orthogonal projection transformation. Then, digital surface model (DSM) data were generated at a 50-cm grid size using ArcMap software (Economic and Social Research Institute, CA). Clumps of dwarf bamboo were extracted as polygon grids from each photograph by visual observation and the projection area was calculated.

**Figure 3:**
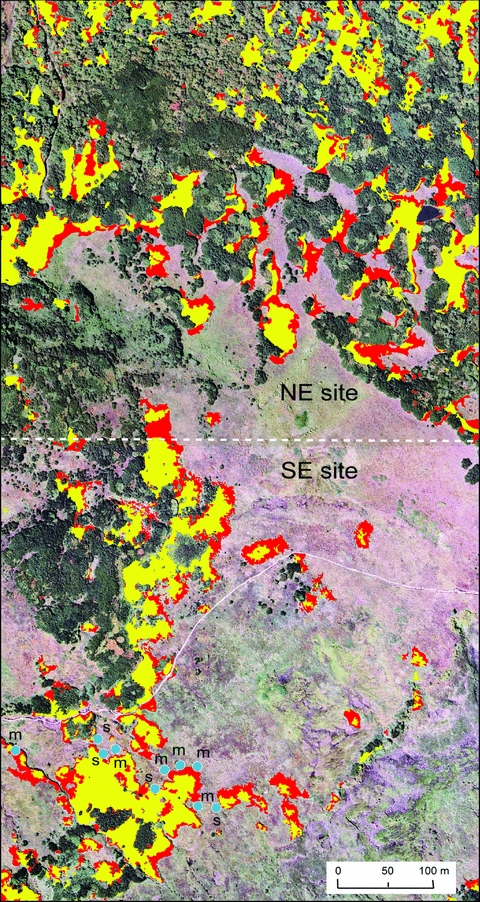
Distribution of dwarf bamboo clumps in the study site. The yellow area indicates the distribution in 1977 and the red area indicates the additional area occupied by dwarf bamboo between 1977 and 2009. Dark green vegetation is*Pinus pumila*clumps that are often located on the western side of dwarf bamboo clumps. Blue circles in the SE site indicate the locations in which the belt-transect survey was conducted. m = diffuse margin, s = distinct margin. Refer Table 1 for the increased and decreased area during this period.

To assess the direction of dwarf bamboo expansion, the distribution of the increased area of dwarf bamboo during 32 years was analyzed within buffer zones of 16-m width from the margin of dwarf bamboo polygons in 1977. The size of buffer zone was decided based on the maximum distance of clump expansion (see Fig. 3). Then, the area of occupation by dwarf bamboo expansion was analyzed in terms of eight aspect components, N, NE, E, SE, S, SW, W, and NW. The area occupied by*P. pumila*clumps was excluded from the analysis because dwarf bamboo did not increase in area toward the resilient*P. pumila*clumps that block a bamboo expansion (see Fig. 3).

The directional preference of dwarf bamboo expansion was analyzed for individual polygons (clumps) by Manly's selectivity index (*w_i_*) as follows:

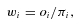
where*o_i_*is the proportion of dwarf bamboo occupation and*π_i_*is the proportion of land area at the azimuthal component*i*within a buffer zone. Then, upper and lower limit of confidence intervals (CI) at the 95% level were calculated for each*i*by the Bonferroni method. Positive preference for a specific azimuthal component was detected when the lower CI was >1, while a negative preference was indicated when the upper CI was <1.

### Field census of dwarf bamboo biomass and development

To quantify the aboveground biomass and culm production of dwarf bamboo, we selected three locations within a large bamboo clump (30 × 40 m^2^) in the SE site as follows: (1) the terminal part was the advancing front, (2) the marginal part was 5- to 10-m inward from the terminal part, and (3) the interior part was 15- to 20-m inward from the terminal part. At each location, we set two 5 × 5 m^2^ plots in 2008 that were divided into 25 individual 1 × 1 m^2^ quadrats, where five quadrats within a central 3 × 3 m^2^ area in each plot were used for measurements. We harvested the aboveground parts of dwarf bamboo in one plot (clipping plot) in late August 2008 at each location. The number of culms per quadrat was counted, culm height for 50 arbitrarily selected culms per plot was measured in which 10 culms of averaged size were selected as a rule of thumb in each of five quadrats, and dry weight of culms was measured after 48 h drying at 80°C in the laboratory. For another plot (control plot) at each location, we recorded the number of culms in late August of 2008, 2009, and 2010. To estimate the speed of advancement of dwarf bamboo, we measured the distance between new culms produced in 2009 and the nearest older culms at the terminal part of this clump for 47 randomly selected culms. This measurement was based on the assumption that new culms might be produced by rhizome extension from the nearest older culms.

### Belt-transect census

**Figure 4:**
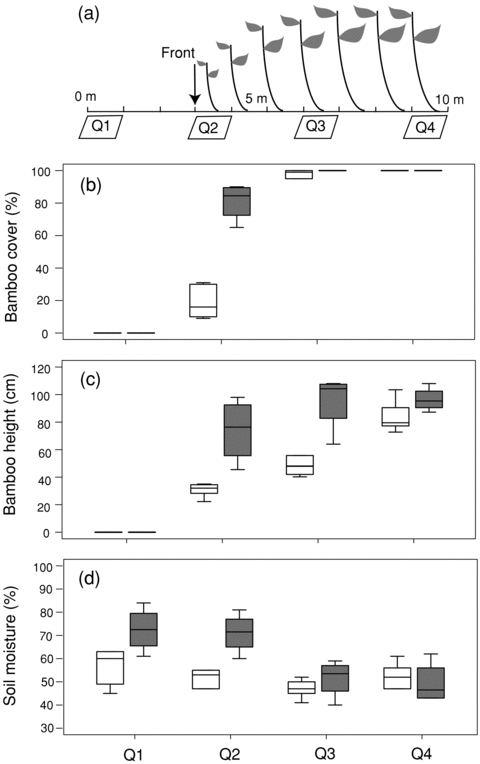
Belt-transect across the margin of dwarf bamboo clumps. (a) A 10-m transect was set from the outside to inside of individual dwarf bamboo clumps in which terminal culms were located at the 3-m point, and 1 × 1 m^2^ quadrats were set at 0–1 m, 3–4 m, 6–7 m, and 9–10 m locations for the measurements of culm performance and the environmental survey. (b) Percent coverage of bamboo canopy in each quadrat. (c) Maximum height of bamboo culms in each quadrat. (d) Soil moisture in volume-metric water content at a depth of 1–12 cm in each quadrat. Open boxes indicate diffuse margins and closed boxes indicate distinct margins. The sample size was 6 for diffuse margins and 4 for distinct margins. Box and whisker plot represents the 75th, 50th, and 25th percentile (boxes) with whiskers from the 90th to 10th percentile.

To determine the pattern of advancement of dwarf bamboo and its impact on the species diversity of snow-meadow vegetation, we conducted a belt-transect census from snow-meadow vegetation to the interior part of dwarf bamboo clumps in the SE site in late August 2008. We selected two types of dwarf bamboo clumps in terms of culm performance of terminal parts. One had a “diffuse margin,” in which bamboo culms gradually changed in density and height from the interior to edge of the clump. The diffuse margins were thought to be advancing into the snow-meadow. The other had a “distinct margin” in which the edge of clump was clear-cut and thought to be stagnant or advancing very slowly. In order to capture typical margin types, we arbitrarily selected six diffuse margin and four distinct margin clumps in late August 2008 (see Fig. 3). We put a 10 × 1 m^2^ belt-transect, in which the 3-m position of the transect was set at the frontier culm of the clump, and set four 1 × 1 m^2^ quadrats at the 0–1 m, 3–4 m, 6–7 m, and 9–10 m positions of the transect (hereafter Q1, Q2, Q3, and Q4, respectively; Fig. 4a). In each quadrat, we measured the culm height and percent coverage of dwarf bamboo, the number of other vascular plant species (species richness), and soil moisture. Volumetric soil water content (%) was measured at six points in each quadrat using a time-domain-reflectometry device (TDR, Hydrosence, Campbell Scientific Inc., QLD, Australia) connected to a 12-cm probe. The mean value of six measurements was used for the analysis. Furthermore, photosynthetically available photon flux density (PPFD) was measured above and below the bamboo canopy using a light quantum measurement device (LI-250, Li-Cor, Lincoln, NE). Then, relative PPFD was calculated to quantify the shading effect of the bamboo canopy.

Transitions of culm height and soil moisture along a belt-transect were analyzed by a linear mixed effect model using the nlme library of R version 2.12, in which quadrat position on a belt-transect (Q1 = 1, Q2 = 4, Q3 = 7, Q4 = 10) and type of margin (diffuse, distinct) were treated as explanatory variables, and the transect as a random factor. Effects of culm height, relative PPFD, and soil moisture on species richness were evaluated using Spearman's rank correlation test.

### Genet identification using microsatellite markers

**Figure 6:**
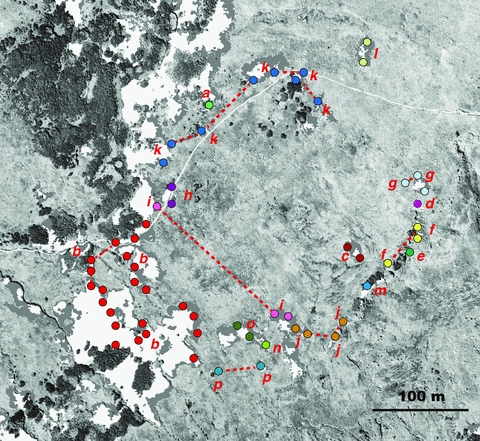
Distribution of clonal patches of dwarf bamboo in the SE site discriminated by the SSR analysis. Separated clumps of the same genotype are connected by broken lines. Dots indicate the sampling points for SSR analysis. For the genetic composition of individual clones (*a*–*p*), refer to Appendix 1.

To clarify the genetic component of dwarf bamboo clumps in the area of advancement, we confirmed genet identification using polymorphic microsatellite markers. Leaf sampling for analysis was conducted in the SE site in 2010. Within a 400 × 400 m^2^ area, leaf samples of 58 culms from 28 clumps were collected and each sampling point was recorded using a handy GPS device (GPSMAP 60CSx, Garmin International Inc., KS; see Fig. 6 for sampling points).

Total DNA was extracted from 100 mg of fresh leaf sample using a DNeasy Plant Mini Kit (Qiagen, Tokyo, Japan), and all microsatellite regions were amplified using a Multiple PCR Kit (Qiagen) as described previously by Kitamura and Kawahara (2009) and Kitamura et al. (2009). Among 14 microsatellite markers examined, the following eight markers were identified with polymorphisms: BWSS4, BWSS7 (Miyazaki et al. 2009), Sasa05G, Sasa06B, Sasa223, Sasa500, Sasa540, and Sasa946 (Kitamura et al. 2009). Genet identification was conducted using these SSR loci.

## Results

### Expansion of dwarf bamboo during a period of 32 years

The area occupied by dwarf bamboo clumps was 10.5% of the total land surface in 1977, while it was 13.3% in 2009, indicating a 26% increase during the 32 years between the aerial surveys (Table 1). The rate of expansion was larger in the SE site (47.5%), where snow-meadow vegetation dominated, compared with the NE site (10.9%). This was because the retreating area of dwarf bamboo during this period was much smaller in the SE site (10.4%) than in the NE site (40.0%; see Table 1 for detail area of changes). The range of advancement reached a maximum of 16 m, corresponding to 50 cm/year. There was a clear trend in the direction of clump advancement (Fig. 3). Dwarf bamboo tended to be distributed on the eastern vicinity of*P. pumila*clumps and they tended to expand in an eastward direction. This is partly because the existence of*P. pumila*clumps blocks the advance of dwarf bamboo clumps. Even when the effect of*P. pumila*was removed, Manly's selectivity index indicated the positive preference for eastern directions (NE, E, SE), while there was a negative preference for other directions (Table 2).

**Table 1:** Occupied area by dwarf bamboo in 1977 and 2009, increased area, decreased area, and rate of expansion during 32 years in the study area

Site	Area in 1977	Area in 2009	Increased	Decreased	Difference
NE site	31,217 m^2^	34,614 m^2^	15,868 m^2^	12,472 m^2^	+10.9%
SE site	21,687 m^2^	31,980 m^2^	12,544 m^2^	2,251 m^2^	+47.5%
Total	52,904 m^2^	66,593 m^2^	28,411 m^2^	14,723 m^2^	+25.9%

**Table 2:** Preference of slope direction for dwarf bamboo expansion. Buffer area denotes an area of 16-m width around the dwarf bamboo patches in 1977 excluding *Pinus pumila* patches, and expansion area indicates the area of dwarf bamboo expansion within the buffer area between 1977 and 2009. Manly's preference index (*w_i_*), standard error, and confidence interval (CI) at the 95% level are shown

Slope	Expansion (m^2^)	Buffer area (m^2^)	*w_i_*	SE	Lower CI	Upper CI	Preference
NE	5,467	26,183	1.124	0.014	1.090	1.159	+
E	6,169	27,590	1.204	0.014	1.170	1.239	+
SE	6,261	32,672	1.032	0.012	1.003	1.061	+
S	3,636	21,591	0.907	0.014	0.871	0.942	–
SW	1,779	10,555	0.908	0.021	0.855	0.960	–
W	1,583	10,019	0.851	0.021	0.799	0.903	–
NW	2,234	14,819	0.812	0.017	0.770	0.853	–
N	3,616	22,135	0.880	0.014	0.845	0.914	–

### Aboveground biomass and culm dynamics of dwarf bamboo

Culm height of dwarf bamboo decreased from 94 cm in the interior part to 24 cm at the terminal part, and the dry mass of culms changed abruptly from 1501 g/m^2^ to 20 g/m^2^ within a 15- to 20-m distance between the clipping plots (Table 3). Culm density decreased from 94.4 per m^2^ at the interior part to 1.2 per m^2^ at the terminal part in 2008. Culm density in each control plot increased from year to year, corresponding to a 30–150% increase (Table 3). Therefore, biomass of dwarf bamboo was actually developing in this clump. At the terminal part, distance of advancement of new culm appearance by the extension of rhizome was 39.3 ± 3.4 SE cm, ranging from 15 cm to 68 cm (*n* = 47).

**Table 3:** Plant height, aboveground biomass, and yearly changes in shoot density of dwarf bamboo at three locations within expanding clumps

Position	Culm height^1^ (cm)	Biomass^1^ (g/m^2^)	Culm density^2^ (m^–2^)		
			2008	2009	2010
Interior part	93.7 ± 2.0 (*n* = 50)	1501	94.4 ± 2.4 (*n* = 5)	114.0 ± 5.0 (*n* = 5)	122.4 ± 6.7 (*n* = 5)
Marginal part	43.0 ± 1.1 (*n* = 50)	355	60.6 ± 3.4 (*n* = 5)	75.4 ± 6.1 (*n* = 5)	83.4 ± 5.4 (*n* = 5)
Terminal part	24.4 ± 0.6 (*n* = 50)	19.7	1.2 ± 0.6 (*n* = 5)	1.6 ± 0.7 (*n* = 5)	3.0 ± 1.23 (*n* = 5)

1 measured in clipping plots in 2008

2 measured in control plots in each year.

### Belt-transect census

The canopy cover of dwarf bamboo at the terminal part (Q2) was contrasted between the diffuse margins (about 20%) and the distinct margins (80%), while bamboo cover at Q3 and Q4 was almost 100% in both types (Fig. 4b). We did not apply the analysis of Linear Mixed Effect model (LME) for bamboo cover because of the large deviations in data distribution. At diffuse margins, culm height decreased gradually from the interior (about 80 cm) to terminal parts (about 30 cm). At distinct margins, culms were tall from the interior to the edges of the plots (100–80 cm, Fig. 4c). LME results revealed that culm height was significantly larger at distinct margins than at diffuse margins (*t* = 3.46, df = 8,*P* = 0.008), culm height then increased toward the interior part (*t* = 6.78, df = 18,*P*< 0.0001), and a significant interaction between margin type and quadrat position (*t* = –3.14, df = 18,*P* = 0.006).

The transition of soil moisture was considerably different between clumps with diffuse and distinct margins (Fig. 4d). Although there was little difference in soil moisture among quadrats in the diffuse type, soil moisture in the distinct type was apparently larger in Q1 and Q2 than Q3 and Q4. LME results revealed that soil moisture was significantly higher for clumps with distinct margins compared with those with diffuse margins (*t* = 2.99, df = 8,*P* = 0.017), soil moisture increased toward the terminal part at a marginal level (*t* = –1.80, df = 28,*P* = 0.083), and a significant interaction between margin type and quadrat position (*t* = –2.10, df = 28,*P* = 0.044). These results indicated that there was a significant difference in soil moisture between the inside and outside of bamboo clumps at distinct margins, while the difference in soil moisture was indifferent across transects at diffuse margins.

**Figure 5:**
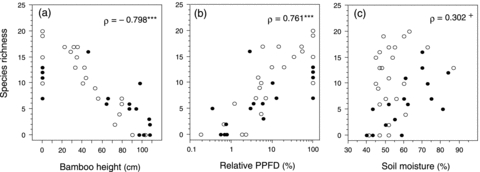
Relationships between species richness and culm height of dwarf bamboo (a), between species richness and relative PPFD (b), and between species richness and soil moisture (c) obtained in the belt-transect survey. Open circles indicate data from diffuse margins and closed circles from distinct margins. ***P< 0.0001, ^†^*P*< 0.10 by Spearman's correlation test.

Species richness clearly decreased from the outside to the interior part of dwarf bamboo clumps: 13.9 species (ranging 7–20) at Q1, 13.3 (5–17) at Q2, 5.9 (0–13) at Q3, and 3.2 (0–7) at Q4. Species richness was negatively correlated with culm height (ρ = –0.798,*P*< 0.0001; Fig. 5a), and positively correlated with relative PPFD (ρ = 0.761,*P*< 0.0001; Fig. 5b). However, correlations between species richness and soil moisture was marginal (ρ = 0.302,*P* = 0.063; Fig. 5c). These results indicate that species diversity is susceptible to shading by dwarf bamboo, but direct effect of soil moisture is small.

### Genet identification

We analyzed 16 SSR loci for 56 culms and found different genotypes in 15 loci (Appendix 1). Fifty-six culms were classified into 16 genotypes that were commonly clustered as clonal patches (Fig. 6). However, some separated clumps were categorized as same genet (such as genet*k*and*i*). The largest clone patch (genet*b*) was >150 m in length. In contrast, small clumps scattered in the eastern part were composed of various genotypes.

## Discussion

Our remote sensing analysis revealed that the distribution area of dwarf bamboo has increased by 48% on a southeast-facing slope during last 32 years. Because the increase in distribution area was mostly caused by the expansion of existing clumps, invasion of dwarf bamboo into the snow-meadows might reflect vegetative growth by branching and extension of the rhizome system. A field census indicated that culm density is increasing from year to year, and the terminal parts of clumps advanced by 39 cm/year, corresponding to a 12.5-m expansion during 32 years. This rough estimate seems to be reasonable from the expansion pattern in this area (Fig. 3).

The present study also revealed that dwarf bamboo expansion is not uniform within a site but highly specific to the slope direction and soil moisture conditions. The rate of increase was larger for a southeast-facing slope (SE site) than a northeast-facing slope (NE site). This difference in rate of increase may reflect the difference in snowmelt time. Due to the strong northwest wind in winter, a large snowpack is formed on the leeward slope, resulting in later snowmelt on southeast-facing slopes. Because the altitudinal distribution center of dwarf bamboo is located in the forest zone, populations in the alpine zone may potentially suffer from a short growing period more or less in terms of thermal limitation. Therefore, late snowmelt may especially restrict growth in the alpine zone due to the short period of time for photosynthesis and tissue formation. As mentioned before, dwarf bamboo basically grows in the leeward vicinity of*P. pumila*clumps because the formation of small-scaled snowdrift patterns by*P. pumila*clumps protects dwarf bamboo from the severe winter climate. The distribution of*P. pumila*is restricted to places with moderate snow cover (Okitsu and Ito 1984). Snow conditions at the NE site may be more suitable for the growth of*P. pumila*compared to the SE site where snowmelt in large parts commonly occurred only in late June to mid-July (G. Kudo, unpublished data). Due to late snowmelt, snow-meadow vegetation is most common in this site. However, snowmelt has been accelerating in snow-meadow habitats in the Taisetsu Mountains, where the day of snow disappearance has been 10 days earlier during the course of the last 20 years (Kudo and Hirao 2006). The extension of the snow-free period may support the invasion of dwarf bamboo into snow-meadows.

The direction of the expansion of dwarf bamboo clumps was found to be predominantly oriented toward southeastern aspects, even when the effect of*P. pumila*was eliminated. This may be related to the early winter microclimate. This side is usually covered with snow already in mid-October, but occasional snowmelt sometimes happens in November. Exposure from snow protection and soil freezing due to little or absent snow cover may cause physiological/physical damage to plants inhabiting snowy habitat (Bokhorst et al. 2009). Because of the formation of snowdrifts at the eastern parts of bamboo clumps, east-facing margins may be better protected from freezing damage in early winter, resulting in larger culm production. To confirm this prediction, the study on the relationship between calm demography and winter environments of microscale habitats are desirable.

Furthermore, clump expansion seemed to be susceptible to soil moisture. In the belt-transect survey, distinct margins, which had not advanced, faced toward wetter soil conditions in comparison with the interior part of clumps. In contrast, diffuse margins, advancing into snow-meadows, did not show a gradient of increasing soil moisture from the interior to outside of clumps. This result indicates that such steady soil moisture conditions may accelerate the expansion of dwarf bamboo clumps into snow-meadows. Alpine vegetation patterns often reflect the microscale variation in soil moisture conditions (Isard 1986), and snowmelt time is an important factor affecting soil moistures (Ostler et al. 1982). An extension of the growing period due to earlier snowmelt may cause increased annual production and biomass of alpine plants (Walker et al. 1994). For example,*Salix herbacea*inhabiting snowbed habitats increased annual shoot growth five times following a 20% extension of growth period (Wijk 1986). This indicates that slight changes in snowmelt time may cause large changes in annual production of plants in late-snowmelt habitats. Therefore, a warming-induced decrease in water content and/or the extension of the snow-free period due to earlier snowmelt may accelerate the expansion of dwarf bamboo distribution into alpine snowbed habitats.

Species richness clearly decreased with the development of dwarf bamboo clumps by responding positively to relative PPFD and negatively to culm height. Species richness was abruptly reduced at places where culm height was taller than 50 cm and relative PPFD was lower than 10%. The number of species decreased to less than one-quarter of the original level by the formation of dense dwarf bamboo clumps in our study. Because an effect of soil moisture on species richness was not detected, species diversity might be reduced by the shading effect of dwarf bamboo. This indicates that snow-meadow species are highly sensitive to competition for light acquisition (Billings 1974).

The biomass of dwarf bamboo was extremely large compared to other alpine vegetation. The aboveground biomass of dense clumps was larger than 1.5 kg/m^2^ and culm density was higher than 100 per m^2^ whose height was often taller than 1 m. A previous study reported that the proportion of belowground to aboveground biomass in*S. kurilensis*changed with developmental stage ranging from 21% to 80% (Yajima et al. 1997). The belowground biomass of 90- to 140-cm tall clumps (similar to our site) was 42% of the aboveground biomass. Therefore, the total dwarf bamboo biomass in our site was estimated as 2.1 kg/m^2^. In alpine ecosystems, the aboveground biomass of developed herbaceous communities is commonly around 100–400 g/m^2^ over a wide range of latitudes (Körner 2003). Therefore, the invasion of dwarf bamboo could have a serious impact on alpine habitats and the growing conditions of other alpine plants, not only through shading effects but also by litter accumulation.

In addition to the expansion of clonal patches, the genotype survey implied the possibility of sexual reproduction in alpine environments. The establishment of new clumps was detected in the 2009 census, although their clump size was very small. Actually, clumps distributed in the central part of snow-meadow vegetation were composed of various genotypes, indicating the possibility of establishment by seed dispersal. Although most dwarf bamboo species are monocarpic plants, recent studies reported that mass flowering is not always common but also sporadic partial flowering might occur frequently (Yamazaki and Nakagoshi 2005; Kitamura and Kawahara 2009). A discontiguous distribution of the same genet across clumps may be evidence of past partial flowering and death within large clones. Sexual reproduction of dwarf bamboo in severe alpine environments, however, is completely unknown and requires further studies.

The present study demonstrated that alpine snow-meadows are a sensitive vegetation type to the invasion of dwarf bamboo supported by a decrease of the snow cover period and by drier soil conditions. Environmental modification through the invasion of dwarf bamboo had a serious effect on low-stature alpine vegetation, because of the large aboveground structure and biomass accumulation, similar as reported in other studies of shrub encroachment (Sturm et al. 2005; Knapp et al. 2008). The performance of dwarf bamboo in alpine ecosystems could be used as a key indicator of global warming impacts on East Asian alpine vegetation.
